# A meta-analysis on depression and subsequent cancer risk

**DOI:** 10.1186/1745-0179-3-29

**Published:** 2007-12-03

**Authors:** Marjolein EJ Oerlemans, Marjan van den Akker, Agnes G Schuurman, Eliane Kellen, Frank Buntinx

**Affiliations:** 1 Maastricht University, Department of General Practice, School of Public Health and Primary Care: CAPHRI, Maastricht, the Netherlands; 2 Catholic University of Leuven, Department of General Practice, Leuven, Belgium

## Abstract

**Background:**

The authors tested the hypothesis that depression is a possible factor influencing the course of cancer by reviewing prospective epidemiological studies and calculating summary relative risks.

**Methods:**

Studies were identified by computerized searches of Medline, Embase and PsycINFO. as well as manual searches of reference lists of selected publications. Inclusion criteria were cohort design, population-based sample, structured measurement of depression and outcome of cancer known for depressed and non-depressed subjects

**Results:**

Thirteen eligible studies were identified. Based on eight studies with complete crude data on overall cancer, our summary relative risk (95% confidence interval) was 1.19 (1.06–1.32). After adjustment for confounders we pooled a summary relative risk of 1.12 (0.99–1.26).

No significant association was found between depression and subsequent breast cancer risk, based on seven heterogeneous studies, with or without adjustment for possible confounders. Subgroup analysis of studies with a follow-up of ten years or more, however, resulted in a statistically significant summary relative risk of 2.50 (1.06–5.91).

No significant associations were found for lung, colon or prostate cancer.

**Conclusion:**

This review suggests a tendency towards a small and marginally significant association between depression and subsequent overall cancer risk and towards a stronger increase of breast cancer risk emerging many years after a previous depression.

## Introduction

Whether or not depression might be a risk factor for developing cancer has long been debated. Reports on the relation between depression and cancer risk are controversial and mixed. Most of these studies are not designed to describe a directional and certainly not a cause and effect relationship. From 1980 onwards several prospective studies have been published and in 1994 a meta-analysis on the subject was conducted [1]. In this meta-analysis the pooled overall odds ratio between depression and subsequent cancer risk was 1.14 (95% confidence interval: 0.99–1.30), which led the authors to conclude to a small and marginally significant association between depression and the subsequent development of cancer. The studies included in the meta-analyses were all published between 1980 and 1990 and possible confounders were not taken into account during pooling. After the publication of this meta-analysis several similar studies were published. We therefore decided to perform a new systematic review to investigate whether the conclusion about depression being a risk factor for cancer development still holds, taking into account the effect of possible confounders and concentrating on general population-based studies only.

## Methods

### Literature search

Our start for selecting studies was the meta-analysis by McGee et al. published in 1994 [1]. The studies included in this meta-analysis were identified and their references were checked for additional relevant publications. We searched Medline, Embase and PsycINFO from 1990 to the end of October 2005 with a highly sensitive search strategy using the keywords depress* in combination with neoplasm* or cancer. Searches were independently performed by three individual researchers of which two are experienced meta-analysts. Their yields were added to one common list of references. Reference lists from identified prospective studies were also checked for other potentially relevant publications not included in the computerized database search and we contacted leading experts in this field as well as researchers we knew to be engaged in recent studies.

### Selection and data collection

Final inclusion was based on the following selection criteria: a prospective, general population-based study, which made use of validated measures of depression as well as questionnaires that resembled Diagnostic Statistical Manual of mental disorders (DSM) criteria for major depression. Studies, in which the diagnosis of depression was based on the subjective judgment of a clinician only, or on the presence of a certain number of symptoms, were not included. We did not use any language restriction. Also publications included in the meta-analysis by McGee et al. [1] were checked according to our own criteria. As a result only four of the seven studies identified by McGee et al. [1] were included in our own meta-analysis.

### Quality assessment

For each study, data were collected on several study characteristics (continent, setting, age range, sex ratio, depression assessment method, method of retrieval of the cancer cases, years of follow-up, type of cancer, and number of cancer patients). Data extraction was performed by one researcher and supervised by at least one senior researcher.

### Analysis

From each study we constructed 2 × 2 tables in order to calculate crude relative risks. If the published study did not provide the data needed for the 2 × 2 table, we tried to contact the corresponding author to complete our tables.

Publication bias was examined by means of a funnel plot. We examined asymmetry visually and measured the degree of asymmetry by using Egger's unweighted regression asymmetry test [2].

For all associations, we examined the presence of heterogeneity visually by inspecting forest plots. Presence of heterogeneity was also quantified. We calculated a chi-square test for homogeneity, an I^2 ^as a measure of the percentage of total variations across studies that is due to heterogeneity rather than chance and the estimate of between studies variance, calculated by comparing the results of fixed and random effect pooling of the same sets of studies. For reasons of readability we only report the I^2 ^as it turned out to be the most powerful in detecting heterogeneity [3]. I^2 ^values of 25%, 50% and 75% are considered to indicate low, moderate and high heterogeneity. To explore reasons for heterogeneity we performed subgroup analyses. As we considered length of the follow-up period and adjustment for smoking behavior as likely being the most important confounders, we included subgroup analyses according to these variables if a sufficient number of studies were available.

Meta-analyses were performed for depression and overall cancer risk and for the risk of individual types of cancer after depression, as long as at least three studies were available. In order to be able to include studies with no depressed cancer case in our pooling, we added 0.5 to each cell of the 2 × 2 table for these studies.

Crude data pooling included all studies for which the crude RR and 95% confidence interval were available or could be calculated. Pooling of adjusted risks included all studies, which were at least adjusted for age. Summary odds ratios and corresponding confidence intervals, were calculated based on random effect modeling. We used the Meta command of STATA version 8.0 software [4] for all calculations except the I^2 ^which was calculated by hand, according to the formula published by Higgins et al [3].

## Results

The review of McGee provided 7 studies [5-11]. After applying our selection criteria we included 4 of these studies [8-11]. One study was not included because only volunteers from a particular workplace [6] were investigated, and one study was excluded because the measuring instrument for depression only produced scores without a cut-point and was not clearly described. Its validity could therefore not be evaluated [5]. The third study was excluded because the depression status at baseline was only based on one single question [7].

The computerized search strategy revealed ten publications from 1990 onwards [12-21]. Furthermore, we found two additional research reports that were only available as reports and not as published papers [22,23].

After applying the selection criteria, another nine studies were included in our meta-analysis. One study was not included because only psychiatric clinic patients were investigated [12]. Another study was excluded because depression was based on the subjective judgment of a clinician [13]. The last study was excluded because the depression status at baseline was only based on one single question [14].

Finally we included data of 13 studies [8-11,15-23] and 127,840 patients in our review. Nine studies provided data on the relation between depression and subsequent overall cancer, of which eight presented sufficient information to enable crude data meta-analysis and seven to enable meta-analysis of adjusted study results. Some of these studies also provided data on subgroups of individual cancer localizations, especially breast, lung, prostate or colon cancer. For studies only provided data on breast cancer and not on overall cancer.

In table 1 the descriptive characteristics of the selected studies are shown. Five studies were conducted in Europe [17,18,21-23], all other studies took place in the USA [8-11,15,16,19,20]. The smallest study involved 1,213 subjects [16], the largest study involved 68,366 subjects [23]. In most studies it was formally stated that cancer-free subjects (either all cancers or specific cancer sites) only were eligible for follow-up [8,9,11,15,17-19,21,23]. For four studies, however, this was not clearly stated [10,16,20,22]. One study only included subjects aged 70 and over [19] and one study only included women aged 56–62[18]. The other studies had a wide range of ages included.

**Table 1:** Descriptive characteristics of included prospective studies on depression and subsequent cancer occurrence

First author (ref)	Total sample (% women)	Setting of cohort	Site of cancer	Age Range	Depression questionnaire	Diagnosis	Follow-up (years)	Number of total cancer patients (% on total)
Hahn (9)	8,932 (100)	Breast cancer-free subjects	Breast only	ns	MMPI depession 70	Medical records and Histology	1969–1982 (13)	120 (1.3%)
Kaplan (8)	6,848 (?)	Population sample, cancer-free subjects	All Lung, Breast Prostate, Colon	"adults"	HPL	Cancer registry	1965–1982 (17)	733 (10.7%)
Zonderman (10)	6,403 (?)	Population sample	All	25–75	CES-D (cut-off score 16) GWB-D (cut off score 13)	Hospitalization records and death certificates	1971–1981 (10)	637 (9.9%)
Linkins (11)	2,264 (?)	Population sample, cancer-free subjects	All	>18	CES-D (depression 16)	Cancer registry and death certificates	1975–1987 (12)	169 (7.5%)
Vogt (20)	1,529 (?)	Population sample	All	≥ 18	DSM-III based questionnaire	Death certificates and state of vital records	1970–1985 (15)	?
Knekt (17)	7,018 (55)	Population sample, cancer-free subjects	All Lung, Breast	30–95	PSE,	Cancer registry	1978–1991 (14)	605 (8.6%)
Penninx (19)	4,825 (64.6)	Population sample, cancer-free subjects	All 11 sites	71–96	CES-D	Hospitalization records and death certificates	1988–1992 (7)	402 (8.3%)
Gallo (15)	2,017 (60)	Population sample, cancer-free subjects	All Lung, Breast, Skin, Colon, Prostate	>18	DIS	Self reports and death certification	1981–1994 (13)	203 (10.1%)
van den Heuvel (22)	2,342 (?)	GP-based	All	All	GP diagnosis ICHPPC-2 criteria	GP-registry	1984–1994 (10)	76 (3.2%)
Schuurman (23)	68,366 (51.2%)	GP-based cancer-free subjects	All Lung, breast, colon, prostate	≥ 20	GP diagnosis ICHPPC-2 criteria	GP-registry	1975–2000 (25)	3,464 (5.1%)
Jacobs (16)	1,213	Population sample	Breast	Mean = 43	DIS	Hospitalization (self report)	1980–1995 (15)	58 (1.1%)
Nyklicek (18)	5,191	Population sample, cancer-free subjects	Breast	56–62	EDS	Cancer registry	1995–2000 (5)	39(3.2%)
Aro(21)	10,892	Population sample, breast cancer-free subjects	Breast	48–50	BDI	Cancer registry	1992–2001 (6–9)	278(2.6%)

MMPI: Minnesota Multiphasic Personality Inventory

DIS: Diagnostic Interview Schedule

HPL: Human Population Laboratory-Depression scale

ICHPPC-2: International Classification of Health Problems in Primary Care

CES-D: Center for Epidemiologic Studies-Depression scale

EDS: Edinburgh Depression Scale

GWB-D: General Well-being schedule, Cheerfull vs Depressed mood scale

BDI: Beck Depression Inventory

PSE: Present State Examination

ns: not specified

Three studies applied the Center for Epidemiologic Studies-Depression (CES-D) scale for measurement of depression[10,11,19]. Other questionnaires used were the Minnesota Multiphasic Personality Inventory (MMPI) [9], the Human Population Laboratory-Depression scale (HPL) [8], the General Well-being schedule, Cheerful vs. Depressed mood scale (GWB-D) [10], the Present State Examination (PSE) [17], the Diagnostic Interview Schedule (DIS) [17], the Beck Depression Inventory (BDI) [21], the Edinburgh Depression Scale (EDS) [18] and the ICHPPC-2 criteria [22,23] which come close to the DSM-IV criteria. In one study questions were used that allowed for a close approximation of the clinical definition of depression according to DSM-III[20].

Three studies had a short follow-up of 4–9 years[18,19,21], while the remainder of studies had between 10 and 25 years of follow-up [8-11,15-17,20,22,23]. The percentage of cancer cases in the study samples varied between 1.1% [16] and 10.7%[8]. In one study this could not be calculated[20]. Definition of cancer was generally based on hospital records, cancer registries, death certificates or a combination of these. Only in one study it was stated that all diagnoses were histologically confirmed[9].

Data for the 2 × 2 tables were complete for seven studies on overall cancer risk [10,11,15,17,19,22,23], for six studies on breast cancer risk [9,15-19,23], and for three studies on lung cancer risk [17,19,23].

### • Overall cancer

In table 2 the crude and adjusted relative risks for subsequent cancer occurrence in patients with and without depression are presented.

**Table 2:** Results from prospective studies on depression and subsequent overall cancer occurrence

First author (ref)	Number of subjects with depression		Number of subjects without depression		Total numbers of subjects	Crude RR (95% CI)	Multivariable Adjusted RR (95% CI) reported in paper	Adjustment factors for multivariable RR
	Cancer	No cancer	Cancer	No Cancer				

Kaplan (8)	117	N.a.	612	N.a.	6,848	-	0.97 males 1.27 females	Age, sex
Zonderman (10)	110	892	527	4,874	6,403	1.13 (0.93–1.37)	1.1 (0.9–1.4)	Age, sex, marital status, smoking, family history of cancer, hypertension, cholesterol level
Linkins (11)	25	343	144	1,752	2,264	0.89 (0.59–1.35)	1.09 (0.69–1.71)	Age
Vogt (20)	N.a.	N.a.	N.a.	N.a.	1,529	1.08 (0.79–1.49)	1.08 (0.77–1.52)	Age, sex, social class, Smoking, duration of health plan membership
Knekt (17)	29	295	486	5,298	7,018	1.07 (0.75–1.52)	0.99 (0.68–1.44)	Age, sex
Penninx (19)	16	130	386	4293	4825	1.33 (0.83–2.13)	1.88 (1.13–3.14)	Age, sex, race, disability, hospital admissions, alcohol, smoking
Gallo (15)	8	82	141	1,338	1,569	0.93 (0.47–1.84)	1.3 (0.6–2.8)	Age, sex, smoking, alcohol
van den Heuvel (22)	9	207	67	2,059	2,342	1.32 (0.67–2.61)	n.a.	
Schuurman (23)	95	1,246	3,369	63,656	68,366	1.41 (1.16–1.72)	1.08 (0.88–1.33)	Age, sex, socioeconomic status

#### Publication bias

Using Egger's unweighted regression asymmetry test we found no evidence for publication bias (p = 0.34).

#### Heterogeneity

Neither visual examination of the forest plot nor the quantitative test provided evidence for heterogeneity of the crude data results (I^2^<0.01).

#### Statistical pooling

Statistical pooling of the eight studies on overall cancer risk after depression revealed an estimated crude summary relative risk (95% CI) of 1.19 (1.06–1.32).

After correction for the confounding factors selected by the original authors (even if this is for age or age and sex only), all seven selected studies reported statistically non-significant associations around the null value [10,11,15,17,20,23] except for one study, which reported a statistically significant association between chronic depression and cancer [19]. Statistical pooling revealed an estimated adjusted summary relative risk of 1.12 (0.99–1.26).

Subgroup analysis on studies that adjusted for smoking or had a follow-up period of ten years or more did not change this picture.

### • Breast cancer

Nine studies reported results for breast cancer separately [8,9,15-19,23] (table 3). Five studies are subgroup analyses of a larger study that also was included in the overall cancer analysis [8,15,17,19,23]. Four studies, however, only examined the association between depression and subsequent breast cancer in females and are not included in the overall cancer analysis [9,16,18,21].

**Table 3:** Results from prospective studies on depression and subsequent breast cancer occurrence

First author (ref)	Number of subjects with depression		Number of subjects without depression		Crude RR (95% CI)	Multivariable adjusted RR (95% CI) reported in paper	Adjustment factors for multivariable RR
	Cancer	No cancer	Cancer	No cancer			

Hahn (9)	15	821	105	7,991	1.38 (0.81–2.37)	1.5 (0.9–2.5)	Age, nulliparity, obesity, hysterectomy
Kaplan (8)	N.a.	N.a.	N.a.	N.a.	-	1.13 (incidence)	Age
Knekt (17)	7	203	47	2,976	2.14 (0.98–4.68)	1.96 (0.88–4.33)	Age
Penninx (19)	0	575	31	3806	0.11 (0.01–1.73)	No depressed cases of malignancy	Age, race, disability, hospital admissions, alcohol, smoking
Gallo (15)	3	N.a.	22	N.a.	3.1 (0.9–11.02)	3.8 (1.0–14.3)	Age, smoking, alcohol
Schuurman (23)	25	830	703	32,746	1.39 (0.94–2.06)	1.06 (0.71–1.58)	Age, socio-economic status
Jacobs (16)	2	9	38	1484	7.28 (2.0–26.52)	17.2 (3.76–77.08)	Age, family history of breast cancer, chronic illness at follow-up, income
Nyklicek (18)	3	837	54	4297	0.29 (0.09–0.92)	0.29 (0.09–0.91)	Family history breast cancer, menopause, oophorectomy, hypothyroidism
Aro (21)						0.70 (0.07–1.63)	Area of residence, age, education, income, children, socioeconomic status, familiy history of breast cancer, smoking, alcohol, physical exercise

Crude relative risks varied widely between 0.11 [18] and 7.28 [16]. It should be mentioned that both studies with a relative risk below 1.0 [18] had the shortest follow-up time (5 and 6 years respectively, while in other studies follow-up times of at least 10 years were used).

#### Publication bias

Using Egger's unweighted regression asymmetry test we found no evidence for publication bias (p = 0.85)

#### Heterogeneity

Both visual examination of the forest plot as well as the quantitative tests suggested heterogeneity of both the crude data (I^2 ^= 0.37) and the adjusted (I^2 ^= 0.37) results.

#### Statistical pooling

Statistical pooling of the seven studies with sufficient crude data for breast cancer resulted in an estimated summary relative risk of 1.46 (0.80–2.64). Eight studies provided adjusted relative risks. The summary relative risk was 1.59 (0.74–3.44). In a sensitivity analysis we excluded the three studies with the smallest follow-up time [18,19,21] and kept the five studies with at least ten years of follow-up. Heterogeneity remained high (I^2 ^= 0.74). All studies had adjusted relative risks above 1.0, and the estimated adjusted summary risk ratio was 2.50 (1.06–5.91). In two studies results were adjusted for smoking. These studies showed an I^2 ^of 0.88 and a summary adjusted relative risk of 1.72 (0.33–9.01).

### • Lung cancer

Five studies reported results on lung cancer after depression [8,15,17,19,23] (table 4).

**Table 4:** Results from prospective studies on depression and subsequent lung cancer occurrence

First author (ref)	Number of subjects with depression		Number of subjects without depression		Crude RR (95% CI)	Multivariable adjusted RR (95% CI) reported in paper	Adjustment factors for multivariable RR
	Cancer	No cancer	Cancer	No cancer			

Kaplan (8)	N.a.	N.a.	N.a.	N.a.	-	1.33 incidence males 1.09 incidence fem	Age, sex
Knekt (17)	4	110	53	2708	1.83 (0.67–4.96)	1.65 (0.60–4.58)	Age, sex (only males)
Penninx (19)	2	144	54	4,625	1.19 (0.29–4.82)	2.10 (0.49–8.92)	Age, sex, race, disability, hospital admissions, alcohol, smoking
Gallo (15)	N.a.	N.a.	N.a.	N.a.	0.7 (0.1–5.1)	1.0 (0.1–7.7)	Age, sex, smoking, alcohol
Schuurman (23)	13	1,328	466	66,559	1.39 (0.81–2.41)	1.25 (0.72–2.17)	Age. Sex, socio-economic status

One study provided a relative risk without a confidence interval [8]. For men and women combined, two studies reported a non-significant positive association[19,23] and one study reported a non-significant negative association[15]. For men only, one study also reported a non-significant positive association[17]. It has to be noticed that the estimated relative risks in most studies were based on very small numbers of depressed patients.

#### Publication bias

Using Egger's unweighted regression asymmetry test we found no evidence for publication bias (p = 0.51)

#### Heterogeneity

Neither visual examination of the forest plot nor the quantitative tests provided evidence for heterogeneity of the crude data results (I^2^<0.01).

#### Statistical pooling

Combining the results of the four studies resulted in an estimated crude summary relative risk of 1.40 (0.90–2.17). After correction for potential confounding factors by the original authors, no statistically significant associations were seen. Statistical pooling revealed an estimated summary relative risk of 1.37 (0.88–2.16), based on four studies [15,17,19,23].

Separate meta-analyses for studies with a follow-up period of ten years or more RR= 1.31; 0.82–2.11) and for studies adjusting for smoking behavior (RR = 1.67; 0.50–5.38) gave comparable results.

### • Colon cancer

Four studies reported results on depression and subsequent risk of colon cancer [8,15,19,23] (table 5). Non-significant changes were reported in three studies [8,19,23]. In the fourth study there were no subjects with colon cancer among the depressives [15].

**Table 5:** Results from prospective studies on depression and subsequent colon cancer occurrence

First author (ref)	Number of subjects with depression		Number of subjects without depression		Crude RR (95% CI)	Multivariable adjusted RR (95% CI) reported in paper	Adjustment factors for multivariable RR
	Cancer	No cancer	Cancer	No cancer			

Kaplan (8)	N.a.	N.a.	N.a.	N.a.	-	0.34 (males) 1.08 (females)	Age, sex
Penninx (19)	N.a.	N.a.	N.a.	N.a.	-	1.37 (0.33–5.74)	Age, sex, race, disability, hospital admissions, alcohol, smoking
Gallo (15)	0	N.a.	19	N.a.	No cases	No cases	Age, sex, smoking, alcohol
Schuurman (23)	14	1,327	568	66,457	1.23 (0.73–2.09)	0.93 (0.55–1.58)	Age, sex, socio-economic status

#### Statistical pooling

Statistical pooling could not be performed due to insufficient data.

### • Prostate cancer

Three studies reported results on depression and subsequent risk of prostate cancer [15,19,23]. (table 6)

**Table 6:** Results from prospective studies on depression and subsequent prostate cancer occurrence

First author (ref)	Number of subjects with depression		Number of subjects without depression		Crude RR (95% CI)	Multivariable adjusted RR (95% CI) reported in paper	Adjustment factors for multivariable RR
	Cancer	No cancer	Cancer	No cancer			

Penninx (19)	N.a.	N.a.	N.a.	N.a.	-	1.47 (1.01–22.79)	Age, race, disability, hospital admissions, alcohol, smoking
Gallo (15)	N.a.	N.a.	N.a.	N.a.	3.6 (0.4–31.3)	11.8 (1–144.3)	Age, smoking, alcohol
Schuurman (23)	3	1338	263	66762	0.57 (0.18–1.78)	0.65 (0.21–2.05)	Age, socio-economic status

#### Publication bias

Using Egger's unweighted regression asymmetry test we found no evidence for publication bias.

#### Heterogeneity

Heterogeneity was tested using the adjusted data results (see table 7), because only two studies presented complete crude data. Both visual examination of the forest plot, and the quantitative tests provided evidence for moderate heterogeneity (I^2 ^= 0.55).

**Table 7:** Relationship between depression and subsequent cancer: summary of the review results

	Number of studies	Summary relative risk (95% CI)	Heterogeneity (I^2^)
Overall cancer			
Crude	8	1.19 (1.06–1.32)	<0.01
Multivariate	7	1.12 (0.99–1.26)	<0.01
Studies adjusting for smoking	4	1.20 (0.97–1.49)	0.23
Studies with follow-up > 10 years	6	1.08 (0.96–1.22)	<0.01

Breast cancer			
Crude	7	1.46 (0.80–2.64)	0.37
Multivariate	8	1.59 (0.74–3.44)	0.37
Studies adjusting for smoking	2	1.72 (0.33–9.01)	0.88
Studies with follow-up > 10 years	5	2.50 (1.06–5.91)	0.74

Lung cancer			
Crude	4	1.40 (0.90–2.17)	<0.01
Multivariate	4	1.37 (0.88–2.16)	<0.01
Studies adjusting for smoking	2	1.67 (0.50–5.38)	<0.01
Studies with follow-up > 10 years	3	1.31 (082–2.11)	<0.01

Prostate cancer (adjusted)	3	1.60 (0.40–6.50)	0.55

#### Statistical pooling

After adjustment for potential confounders three studies reported an increased cancer risk [15,19], one found a non-significant decreased risk [23]. Statistical pooling revealed an estimated summary adjusted relative risk of 1.60 (0.40–6.50).

### • Other cancers

Two studies reported results on depression and subsequent risk of skin cancer [15,19] and two studies reported results on depression and subsequent risk of non-prostate urinary tract cancer [15,19,23]. All reported a non-significant association.

For no other cancer localization more than one study provided results.

## Discussion

### • Results

We summarized study results from 13 prospectively designed and general-population-based studies on depression and the subsequent risk of cancer. For overall cancer, statistical pooling revealed a summary relative risk (95%CI) of 1.19 (1.06–1.32) at crude data analysis (based on eight studies with complete data) and 1.12 (0.99–1.26) after adjustment for potential confounders (seven studies). Five studies adjusted for more possible confounders than age and sex only [10,15,19,20,23]. These studies gave a similar result (summary relative risk = 1.14; 0.99–1.31). Also subgroup analyses including only studies adjusting for smoking (1.20) or studies with a follow-up of ten years or more (1.09) gave similar and non-significant summary relative risks. One of the eight available studies could not be included in our pooled estimate of adjusted overall cancer risk after depression because of insufficient information [8]. However, inclusion of this study would probably not have resulted in a different summary relative risk since it reported an association that was near to our pooled result.

No significant association was found between depression and subsequent breast cancer risk, based on seven heterogeneous studies, with or without adjustment for possible confounders. Subgroup analysis on studies with and without adjustment for smoking behavior did not change the picture. Subgroup analysis with a follow-up time of ten years or more, however, resulted in a statistically significant summary relative risk of 2.50, which would be a strong extra risk.

No significant associations were found for lung, colon or prostate cancer.

### • Evidence from other studies

The results of our meta-analysis suggest a small but increased risk for overall cancer after depression. This is consistent with the marginally significant association (1.14; 0.99–1.30) that was earlier described in the previous review by Mc Gee et al in 1994 [1]. They included far less studies than in our review, some of their studies were not general population-based, they did not perform any of the subgroup analyses we performed and they did not analyze the association with individual cancer locations.

### • Review limitations

No less than ten different questionnaires were used for the measurement of depression in the 13 studies. This problem adds to the multiple conceptual problems concerned with the definition of depression[24]. Since there were only three studies in which the same questionnaire was used, we could not stratify results according to the measurement instrument that was used to diagnose depression. However, differences in reported results may originate from the use of these different instruments.

It is possible that our findings result from random error. After all we performed a lot of tests. However, our results seem plausible and consistent and even the non-significant results point in the same direction. Of all individual study results on overall and breast cancer that we used for pooling only three had an adjusted relative risk below 1.0. Between-study heterogeneity may also result from differences in the sets of potential confounding factors that have been adjusted for in multivariable analyses. In some cases we are not sure that the list of co-variables that were reported in the multivariate model, were the complete set of variables that was collected. We cannot exclude that more variables were stepwise removed during model building and that only significant ones were reported.

On the basis of the data reported in table 1, we roughly estimated the yearly cancer incidence rates per study. For overall cancer they range between 2 and 12 per 1,000 patient-years. Although this is a broad range, they seem to follow a Gaussian curve and there are no real outliers. For the four studies which focus on breast cancer only, the rates are closer to each other (1 to 6 per 1,000 patient-years).

Neither for overall cancer nor for breast cancer risk we have reason to expect that our results are biased by one study with an extreme high or low relative risk. For overall cancer, all results are quite near to each other and for breast cancer the studies with both the highest [16] and lowest [18] relative risk have low to moderate sample sizes. In both cases, the study with the highest sample size [23] tends to decrease the magnitude of the association. In case this study would be to influential, the 'real' association would therefore even be larger.

### • Mechanisms explaining association

We can only speculate about mechanisms explaining a possible association between depression and subsequent risk of cancer. Risk factors for cancer tend to be active long before the occurrence of the first signs. This complies with our finding that the increased breast cancer risk only became apparent in studies with a follow-up of ten years or more. At the other hand it may be possible that depression is an indicator or a consequence of other changes in the body that are the first steps in the oncogenesis. A similar pattern was found in the positive association between herpes zoster and subsequent cancer in older people that only emerges after the first year and increases with time [25], and between postmenopausal hormone therapy and breast cancer after four years[26].

It has been suggested that depression affects the immune and hormonal system [27]. It may so alter the body's defense systems against cancer. In the past an increased risk of Parkinson's disease after depression was suggested to be associated with a common etiological base [28]. However, in our review the studies with a short follow-up time reported no increased risk of depression for developing cancer.

It could also be hypothesized that both depression and increased cancer risk could be related to the presence of one or more genetic characteristics that may be either common or very nearly located in the genome. For the time being, we have no evidence, however, to support this latter hypothesis.

It could also be argued that women experiencing depression at early ages are less likely to have a large number of children, thereby increasing their breast cancer risk.

Alcohol intake [29] and smoking [30] may have an effect on the relation between depression and cancer risk. Such effect may either be called confounding or a step in the etiological path from depression resulting in increased smoking or drinking and finally increased cancer risk. However, in our review we did not find any significant difference between studies that adjusted for smoking and those that did not.

In the past, experimental studies have suggested that antidepressant drugs may increase cancer risk or promote tumor growth [31,32]. Lawlor et al. performed a systematic review on the epidemiologic and trial evidence of an association between antidepressant drugs and breast cancer. Pooled data from 31 primary efficacy drug company trials of fluoxetine suggested no increased risk, but the short duration of these trials may have been insufficient to detect an association [33]. The authors also included 5 cohort studies. One prospective study found an increased breast cancer risk after adjustment for a number of potential confounding factors. The other studies reported no significant association. In 2003 another review collected data from six studies on antidepressant drugs and breast cancer risk. Several studies reported that certain antidepressant drugs may be associated with a slightly increased breast cancer risk, however literature was inconsistent. Methodological limitations of these studies include lack of adjustment for potential confounders, lack of information on duration of use and limited sample sizes [34]. The most recent review we found suggests that antidepressant use might increase the risk of breast cancer, because some psychotropic drugs raise prolactine levels and some specific antidepressants acted as tumor promoters in rodents. However, similar to other reviews also this one doesn't report an increase risk of breast cancer after the use of antidepressants [35]. So, with the evidence available at this moment, it is difficult to disentangle the possible effects of depression and antidepressants on the occurrence of subsequent cancer. It therefore will also be difficult to translate these results in preventive interventions.

Summarizing, we believe that this review presents a tendency towards a small and marginally significant association between depression and subsequent overall cancer risk and forwards a stronger increase of breast cancer risk emerging many years after a previous depression.
